# Diagnostic performance of machine learning applied to texture analysis-derived features for breast lesion characterisation at automated breast ultrasound: a pilot study

**DOI:** 10.1186/s41747-019-0121-6

**Published:** 2019-11-01

**Authors:** Magda Marcon, Alexander Ciritsis, Cristina Rossi, Anton S. Becker, Nicole Berger, Moritz C. Wurnig, Matthias W. Wagner, Thomas Frauenfelder, Andreas Boss

**Affiliations:** Institute of Diagnostic and Interventional Radiology, University Hospital Zurich, Raemistrasse 100, 8091 Zurich, Switzerland

**Keywords:** Breast neoplasms, Machine learning, Ultrasonography

## Abstract

**Background:**

Our aims were to determine if features derived from texture analysis (TA) can distinguish normal, benign, and malignant tissue on automated breast ultrasound (ABUS); to evaluate whether machine learning (ML) applied to TA can categorise ABUS findings; and to compare ML to the analysis of single texture features for lesion classification.

**Methods:**

This ethically approved retrospective pilot study included 54 women with benign (*n* = 38) and malignant (*n* = 32) solid breast lesions who underwent ABUS. After manual region of interest placement along the lesions’ margin as well as the surrounding fat and glandular breast tissue, 47 texture features (TFs) were calculated for each category. Statistical analysis (ANOVA) and a support vector machine (SVM) algorithm were applied to the texture feature to evaluate the accuracy in distinguishing (i) lesions *versus* normal tissue and (ii) benign *versus* malignant lesions.

**Results:**

Skewness and kurtosis were the only TF significantly different among all the four categories (*p* < 0.000001). In subsets (i) and (ii), a maximum area under the curve of 0.86 (95% confidence interval [CI] 0.82–0.88) for energy and 0.86 (95% CI 0.82–0.89) for entropy were obtained. Using the SVM algorithm, a maximum area under the curve of 0.98 for both subsets was obtained with a maximum accuracy of 94.4% in subset (i) and 90.7% in subset (ii).

**Conclusions:**

TA in combination with ML might represent a useful diagnostic tool in the evaluation of breast imaging findings in ABUS. Applying ML techniques to TFs might be superior compared to the analysis of single TF.

**Electronic supplementary material:**

The online version of this article (10.1186/s41747-019-0121-6) contains supplementary material, which is available to authorized users.

## Key points

- Analysis of texture features on automated breast ultrasound can help to categorise imaging findings.
- Machine learning can be applied to texture features to categorise breast lesions.
- Machine learning performs better than the analysis of single texture features.

## Background

In women with dense breast tissue, the combined use of mammography and hand-held ultrasound (HHUS) in breast cancer screening boosts breast cancer detection rate with additionally detected 2–4 cancers per 1,000 women [1–4]. However, the use of HHUS in the screening setting remains controversial due to its inherent limitations including the lack of standardisation and the necessary level of operator experience [4, 5]. In recent years, automated breast ultrasound (ABUS) has been introduced to overcome some of HHUS limitations. ABUS provides technique standardisation via the acquisition of standardised views as well as scanning parameters and resolves the issue of operator subjectivity and variation [6]. Nevertheless, interpretation of imaging findings remains highly dependent on reader skills and experience. Standardised acquisition in terms of scanning parameters (*e.g.,* focus, gain) offers the opportunity to apply tools for image analysis that can support the characterisation of imaging findings.

Texture analysis (TA) is an integral part of the emerging field of radiomics and allows a quantitative and objective assessment of tissue heterogeneity by evaluating the distribution and relationship of pixel or voxel grey levels in the image [7, 8]. In most of the cases, methods based on statistical analysis are used to represent the interdependence of grey-level values. TA applied to computed tomography and magnetic resonance imaging has already shown promising results in predicting pathologic features, prognosis and response to therapy for various diseases and body compartments and can potentially be used in ABUS imaging for lesion analyses [9–16]. Moreover, machine learning (ML) can be applied to data from TA such that algorithms are trained to learn specific patterns and categorise the imaging findings [17].

In this context, the primary purpose of our study was to determine if features derived from TA can be used to distinguish normal tissue, malignant and benign solid lesions in ABUS. Second, we evaluated whether ML applied to TA data can accurately categorise ABUS findings. Third, we compared ML to the analysis of single texture features to categorise ABUS finding based on TA.

## Methods

### Study subjects

The local ethics board approved this retrospective study (“Kantonale Ethikkommission Zurich”; Approval Number: 2016-00064). The need for informed consent was waived. Between December 2015 and June 2017, all women with at least one histologically proven malignant lesion (*n* = 27; median age 54 years; range 30–85 years) who underwent ABUS imaging were identified from the hospital database (University Hospital Zurich). An equal number of women (*n* = 27) with at least one benign solid lesion (median age 44 years; age range 27–73 years) who underwent ABUS during the same study period were also included. In case of a malignant lesion, the histological type was collected. All benign solid lesions had to be either histopathologically proven fibroadenomas or stable lesions with a follow-up of at least 24 months. ABUS was performed in addition to mammography in 39 women with American College of Radiology breast density category *c* or *d* [18] undergoing screening examination and as unique imaging examination in 15 women younger than 40 years undergoing routine controls. None of the patients was symptomatic or had strong family history of breast cancer (*i.e.,* no BRCA1 or BRCA2 mutation carriers, no first-degree relatives of BRCA1 or BRCA2 mutation carriers, and no women with three or more events of ovarian cancer or male breast cancer or breast cancer in women younger than 60 years in first- or second-degree relatives in either maternal or paternal line). The maximum diameter in ABUS was annotated for all lesions.

### ABUS examination

Images were acquired with ABUS (Invenia™ Automated Breast Ultrasound System, General Electric Healthcare, Sunnyvale, CA, USA) using a C 15-6XW reverse curve, 5–14 MHz transducer with an aperture length of 15.3 cm, a transducer travel distance of 16.9 cm, and a depth up to 5 cm. An abundant layer of water-based lotion is applied to the breast in order to maximise the coupling between the transducer and the skin. The standard acquisition included three volumes per breasts, so-called anteroposterior, lateral, and medial in order to guarantee coverage of the entire breast. Slices had a thickness of 0.5 mm. Volume acquisitions were performed in the axial plane, and the 3D reconstructions in the sagittal and coronal planes were automatically provided using a dedicated workstation.

### Image selection and texture analysis

All axial images encompassing the lesion in the three volumes were analysed separately. Images in which the visibility of the lesion was altered because of artefacts (*i.e.,* inadequate compression during the volume acquisition or inadequate lotion with impaired acoustic coupling at the contact surface between the transducer and the skin) were excluded from the analysis (*n* = 63). These images were in general only part of a patient examination (*e.g.,* two to three images in one of the volumes) and did not determine any complete exclusion of patients. Normal fat and fibroglandular tissue were evaluated in two additional, arbitrarily selected images for each patient, usually from the upper outer quadrant (in patients with malignant lesions in the contralateral breast) in order to evaluate the texture features of normal breast tissue. The image selection was performed by a radiologist with 8 years of experience in breast imaging and 3 years of experience in ABUS imaging.

TA was performed in MATLAB (v2016b, The MathWorks Inc., Natick, MA, USA) with an established routine-based procedure, as already described [19, 20]. A region of interest (ROI) was drawn freehand by a radiologist (with 8 years of experience in breast imaging) who delineated the outer edge of the lesion or the maximal continuous area of fibroglandular or fat tissue included in a single image. A second radiologist (with 7 years of experience in breast imaging) performed the same evaluation in five benign and five malignant lesions. In order to minimise intrascanner effects, ROI content normalisation between the mean and three standard deviations (*μ* ± 3 *σ*) was performed as a first step of the TA [21, 22]. Subsequently, 47 features were computed [9] (Table 1). The first order features (entropy, variance, skewness and kurtosis) were directly extracted from the histogram of all grey levels in the ROI. The second and high-order features were derived from the respective grey-level matrices (*i.e.,* grey-level co-occurrence matrix [GLCM]; grey-level run length matrix [GLRLM] and grey-level size zone matrix [GLSZM]) and included more information concerning grey-level distribution by accounting for the relative position of each pixel with respect to the other pixels of the image [9, 23].

**Table 1:** First order and second and high order texture features

Histogram-derived	GLCM	GLRLM	GLSZM
Entropy	Contrast	Short-run emphasis (SRE)	Small zone emphasis (SZE)
Variance	Correlation	Long-run emphasis (LRE)	Large zone emphasis (LZE)
Skewness	Energy	Grey-level non-uniformity (GLN)	Grey-level non-uniformity (GLN)
Kurtosis	Homogeneity	Run length non-uniformity (RLN)	Zone-size non-uniformity (ZSN)
Contrast	Short-run emphasis (SRE)	Run percentage (RP)	Zone percentage (ZP)
Correlation	Long-run emphasis (LRE)	Low grey-level run emphasis (LGRE)	Low grey-level zone emphasis (LGZE)
Energy	Grey-level non-uniformity (GLN)	High grey-level run emphasis (HGRE)	High grey-level zone emphasis (HGZE)
Homogeneity	Run length non-uniformity (RLN)	Short-run low grey-level emphasis (m_SRLGE)	Small zone low-grey level emphasis (SZLGE)
	Run percentage (RP)	Short-run high grey-level emphasis (SRHGE)	Small zone high grey level emphasis (SHZGE)
	Low grey-level run emphasis (LGRE)	Long-run low grey-level emphasis (LRLGE)	Large zone low grey-level emphasis (LZLGE)
	High grey-level run emphasis (HGRE)	Long-run high-grey level emphasis (LRHGE)	Large zone high grey-level (LZHGE)
	Short-run low grey-level emphasis (SRLGE)		Grey-level variance (GLV)
	Short-run high grey-level emphasis (SRHGE)		Zone-size variance (ZSV)
	Long-run low grey-level emphasis (LRLGE)		
	Long-run high grey-level emphasis (LRHGE)		

*GLCM* Grey-level co-occurrence matrix, *GLRLM* Grey-level run length matrix, *GLSZM* Grey-level size zone matrix

### Machine learning

#### Data preparation

Preprocessing and preparation of the dataset for ML were performed with routines written in Python and Scikit-learn (www.scikit-learn.org, release 0.18.1). All features obtained from texture analyses were standardised for the whole dataset using the Scikit-learn-embedded “StandardScaler” class, by removing the mean and scaling the data to unit variance. To account for multiclass classification, the dataset with four classes (malignant lesions, benign solid lesions, fat tissue, glandular tissue) was split into two balanced sub-datasets, each consisting of two classes: (i) solid lesions *versus* normal fat and glandular tissue and (ii) malignant lesions *versus* benign solid lesions. To measure the unbiased performance of the classifier each sub-dataset was randomly shuffled and split in a stratified manner into training and validation partition, with a ratio of 0.8–0.2. The validation partition was excluded from the training process, serving as “unseen” real-world data. Thereby, special attention was put on the fact that each TA dataset in each validation partition was acquired from an individual patient.

#### Support vector machine classifier

An ML model based on the support vector machine (SVM) algorithm with radial basis decision function and fivefold cross-validation was implemented using Scikit-learn. In order to determine the optimal hyperparameters for the SVM, a nested grid search on each fold was implemented by specifying the parameter for gamma and *C* in a logarithmic scale from 0.00001 to 0.001 and 1 to 1,000, respectively. On the training partition, for each sub-dataset, the mean cross-validation accuracies of the classifier for each combination of the specified parameter value was calculated from each fold and depicted as heatmap as a function of *C* and gamma. The parameter combination reaching the highest validation accuracy for the corresponding sub-dataset was chosen for the classification task on the test dataset.

#### Feature selection

To select the reduced feature set (RFS) of optimal features with superior discriminative power from the full feature set (FFS), a recursive feature elimination with cross-validation (RFECV) was performed on each of the sub-datasets. Thereby, each individual feature was ranked and the best set of features according to the classification accuracy was selected. This selection process initially included all 47 features of the dataset and then gradually removed with each iteration of those features, which contributed least to improve the classifier performance. The feature ranking was generated with regard to the number of iterations when the corresponding feature was removed and an optimal number of features was determined [24]. Subsequently, the three previously defined data subsets in the training and validation partition were reduced to the RFECV obtained optimal features, and the SVM classifier was trained and tested again on the RFS applying the same preprocessing steps and hyperparameter tuning as for the FFS.

### Statistical analysis

Normally distributed data are reported as means with standard deviations otherwise as median and interquartile range (IQR). Normal distribution was assessed by using the Kolmogorov-Smirnov test. A one-way analysis of variance was performed for comparison of all texture features among malignant lesions, benign solid lesions and fat and fibroglandular tissue with post hoc Bonferroni correction (only *p* values less than 0.0001 were considered significant). Unpaired *t* test was used to compare all texture features between lesions (benign and malignant) *versus* normal tissue (fibroglandular and fat tissue). The receiver operating characteristic (ROC) curve was computed in the case of features with significant differences. The linear relationship between the different texture features in the FFS was graphically reported via a correlation matrix. For each data subset and corresponding set of features (FFS, RFS) of the validation partition, the overall and tissue-specific performance of the SVM classifier were quantified in terms of classification accuracy and metrics of the confusion matrix [25]. From the generated classification probabilities and confusion matrices, sensitivity and 1-specificity were extracted, and the area under the curve (AUC) was calculated. AUCs were compared with each other according to DeLong’s non-parametric test using MedCalc for Windows, version 18.2.1 (MedCalc Software, Osten, Belgium). A *p* value of less than 0.05 was considered for significance. The inter-reader agreement for the different TA features was evaluated using the intraclass correlation coefficient (ICC) and interpreted according to the criteria by Landis and Koch [26]: an ICC of 0.41–0.60 indicated moderate agreement, an ICC of 0.61–0.80 indicated substantial agreement and 0.81–1.0 indicated almost perfect agreement. All statistical analyses were performed with commercially available software (SPSS, release 22.0; SPSS Inc, Chicago, IL, MedCalc for Windows and d the Scikit-learn package with Python release 3.6) [27].

## Results

Thirty-eight solid benign solid lesions (5 biopsy-proved fibroadenomas) and 32 malignant lesions (30 invasive ductal carcinomas, 2 invasive lobular carcinomas) were evaluated in 54 women. Nine patients had multiple benign lesions, three patients had multifocal, and one patient multicentric disease. The median maximum diameter of benign lesions was 14 mm (IQR 12.0–18.0 mm, range 7–36 mm) and of malignant lesions was 14 mm (IQR 10.5–19.8 mm, range 5–50 mm). A total of 253 images from malignant (approximately 7 images/lesion, range 2–16), 254 images from benign lesions (approximately 6 images/lesion, range 3–16) and 108 images each for fat and fibroglandular tissue were analysed.

### Texture analysis

**Fig. 1:**
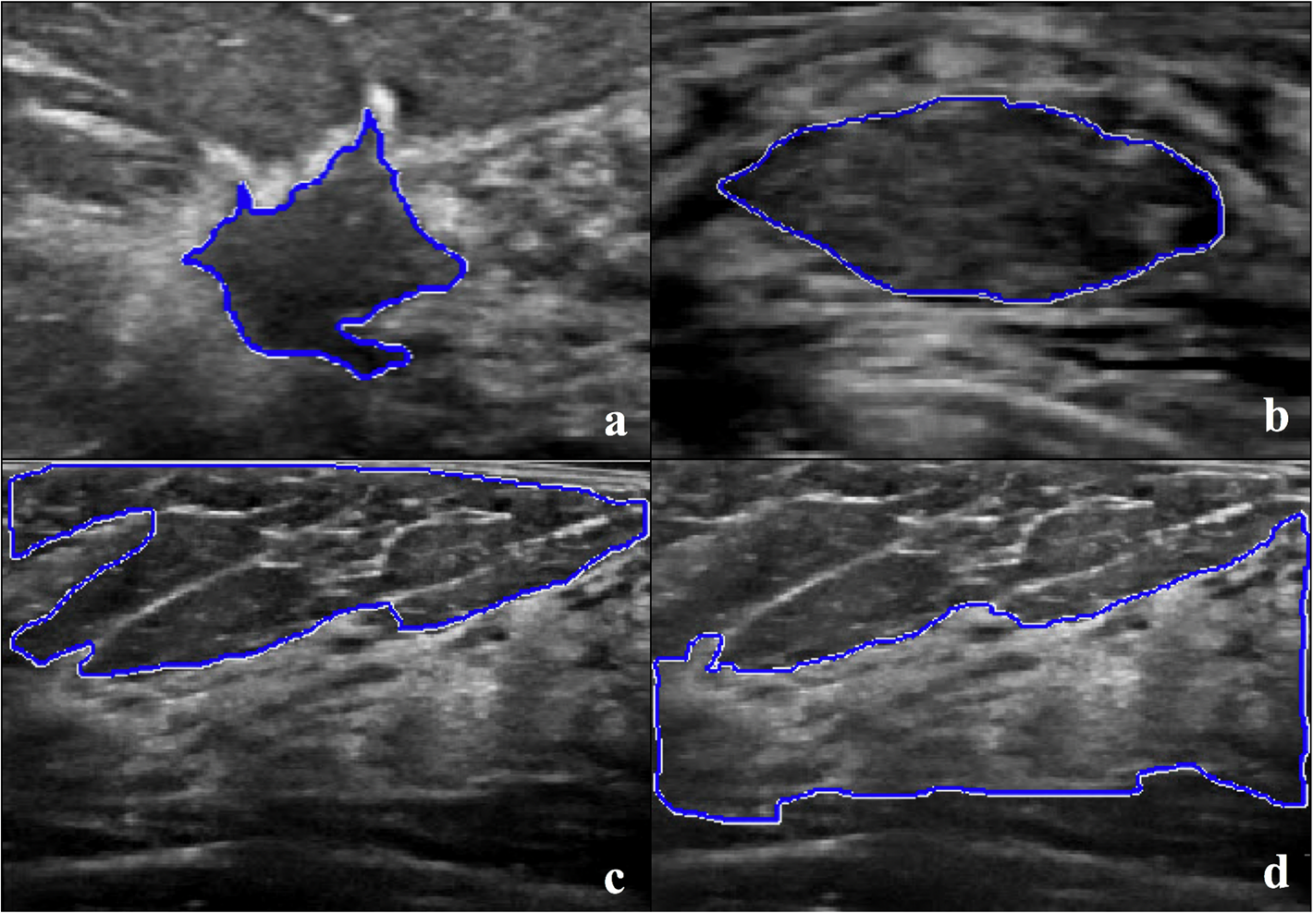
Axial images obtained with automated breast ultrasound (ABUS). A region of interest was drawn freehand marking the outer edge of the lesion or the maximal continuous area of fat or fibroglandular tissue included in the image. Invasive ductal carcinoma in a 53-year-old patient undergoing screening mammography and ABUS (**a**). Stable benign solid lesion after a 48-month follow-up in a 35-year-old woman undergoing routine control (**b**). Fatty tissue (**c**). Fibroglandular tissue (**d**)

**Fig. 2:**
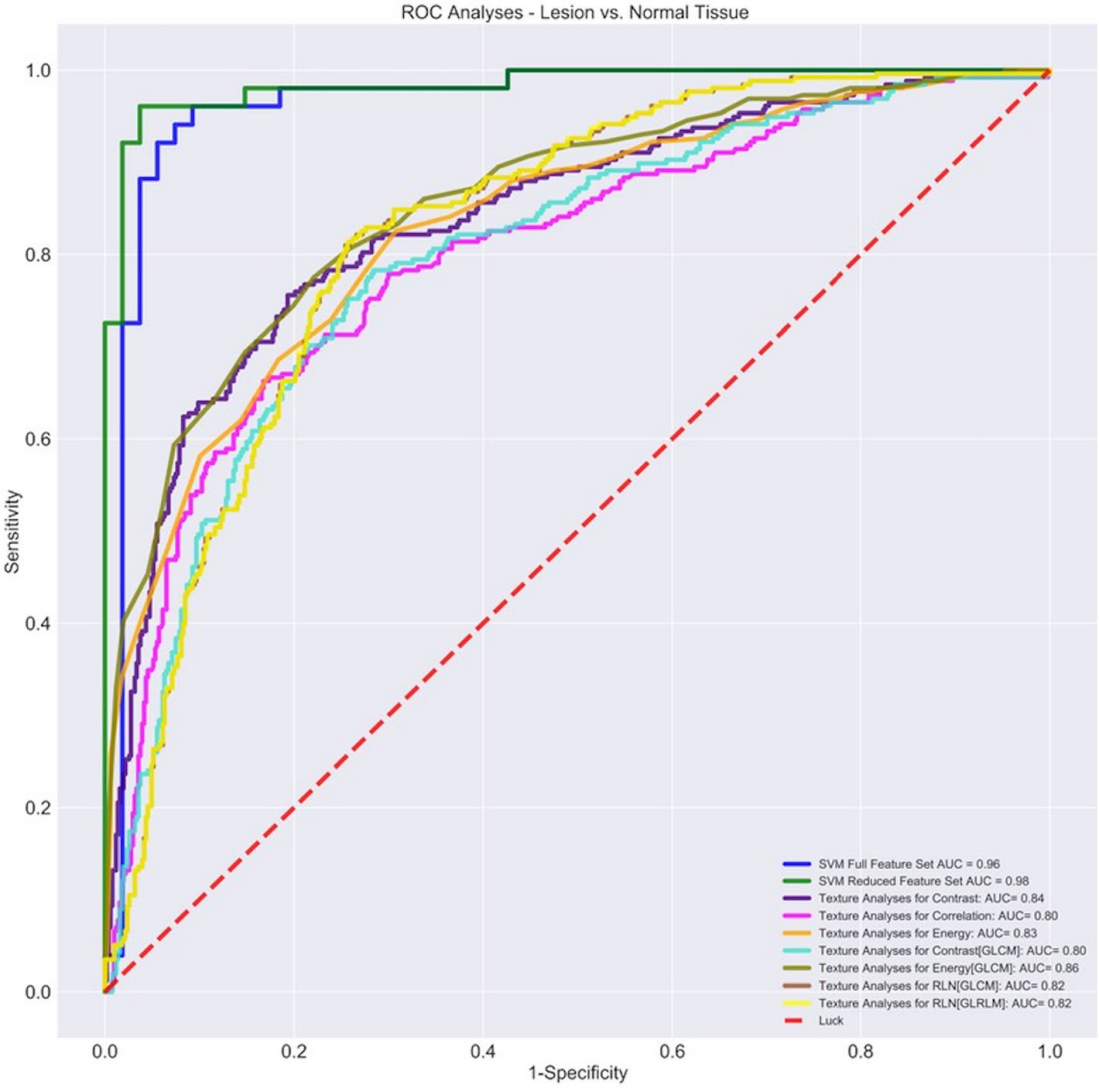
Receiver operating characteristic (ROC) curves of texture analysis features with the highest area under the curve values (Table 2) as well as ROC curves obtained with machine learning support vector machine (SVM) algorithm with full and reduced features when used to compare lesions *versus* normal tissue on automated breast ultrasound

**Fig. 3:**
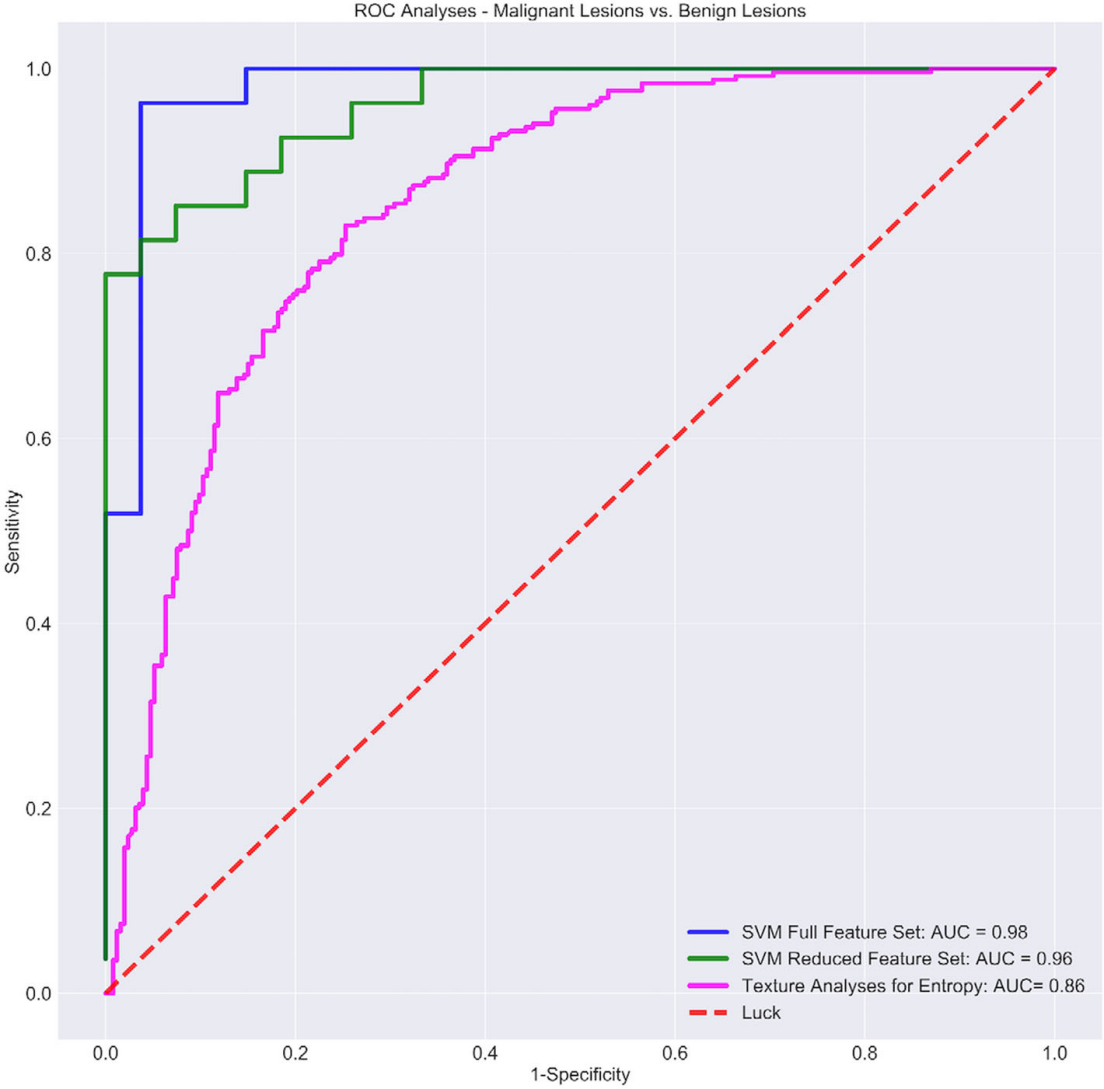
Receiver operating characteristic (ROC) curve obtained from the texture analysis for entropy as well as ROC curves obtained with machine learning support vector machine (SVM) algorithm with full and reduced features when used to compare malignant *versus* benign solid breast lesions on automated breast ultrasound

Median ROI size was 1,312 pixels (IQR 1,161–2,461) for benign lesions, 2,220 pixels (IQR 1,638–2,839) for malignant lesions, 10,529 pixels (IQR 8,074–15,205) for fatty tissue, and 14,296 pixels (IQR 12,736–19,845) for fibroglandular tissue (Fig. 1a–d). Skewness and kurtosis were the only features significantly different among the four categories (*p* < 0.000001). Texture features, which exhibited significant differences when comparing lesions *versus* normal tissue and malignant *versus* benign lesion, with corresponding AUC, are reported in Tables 2 and 3 as well as in Figs. 2 and 3, respectively. At the ROC analysis, the energy was the texture feature with the maximum AUC value in the comparison of lesions *versus* normal tissue (0.86, 95% CI 0.82–0.88) and a total of seven features had AUC values equal or superior to 0.80. Entropy was the texture feature with the maximum AUC value (0.86, 95% CI 0.82–0.89) in the comparison between benign *versus* malignant lesions and the only one with an AUC value superior to 0.80. The ICC showed substantial to an almost perfect agreement in the measure of all texture features (ICC = 0.65–0.96, Additional file 1: Table S1).

**Table 2:** Texture features that showed significantly different mean values comparing lesions (benign and malignant) *versus* normal tissue (fat and fibroglandular) and corresponding area under the curve (AUC)

Feature	Lesions (mean ± standard deviation)	Normal tissue (mean ± standard deviation)	*p* value	AUC (95% confidence interval)
Entropy	5.48 ± 0.35	5.65 ± 0.24	< 0.00001 for all	0.67 (0.63–0.71)
Variance	153.53 ± 23.59	137.75 ± 20.12		0.70 (0.66–0.73)
Contrast	24.10 ± 9.87	40.10 ± 12.5		0.84 (0.81–0.87)
Correlation	0.88 ± 0.06	0.81 ± 0.06		0.80 (0.65–0.83)
Energy	3.4 × 10^−3^ ± 1.5 × 10^−3^	2.2 × 10^−3^ ± 0.7 × 10^−3^		0.83 (0.80–0.86)
Homogeneity	0.36 ± 0.05	0.33 ± 0.04		0.72 (0.69–0.76)
Contrast	26.43 ± 12.38	40.94 ± 13.41		0.80 (0.76–0.83)
Correlation	0.87 ± 0.06	0.81 ± 0.07		0.78 (0.74–0.82)
Energy	3.9 × 10^−3^ ± 1.4 × 10^−3^	2.6 × 10^−3^ ± 0.7 × 10^−3^		0.86 (0.82–0.88)
Homogeneity (GLCM)	0.36 ± 0.05	0.33 ± 0.04		0.71 (0.66–0.74)
GLN (GLCM)	78.42 ± 77.69	156.28 ± 104.76		0.79 (0.76–0.83)
RLN (GLCM)	1,870.43 ± 1,792.01	4,085.00 ± 2,628.83		0.82 (0.79–0.85)
LRHGE (GLCM)	1,706.53 ± 209.23	1,635.50 ± 197.26		0.61 (0.57–0.65)
SRE (GLRLM)	0.91 ± 0.02	0.92 ± 0.04		0.69 (0.65–0.73)
LRE (GLRLM)	1.47 ± 0.17	1.39 ± 0.12		0.65 (0.61–0.69)
GLN (GLRLM)	78.53 ± 77.67	156.36 ± 104.73		0.79 (0.76–0.83)
RLN (GLRLM)	1,871.42 ± 1,786.51	4,080.90 ± 2,622.01		0.82 (0.79–0.85)
LRHGE (GLRLM)	1,677.30 ± 196.74	1,598.70 ± 168.52		0.61 (0.57–0.65)
LZE	5.38 ± 3.34	4.07 ± 2.38		0.66 (0.62–0.70)
HGZE	1,221.08 ± 45.52	1,191.37 ± 86.03		0.67 (0.63–0.71)

*GLCM* Grey-level co-occurrence matrix, *GLN* Grey-level non-uniformity, *RLN* Run length non-uniformity, *LRHGE* Long-run high grey-level emphasis, *GLRLM* Grey-level run length matrix, *SRE* Short-run emphasis, *LRE* Long-run emphasis, *LZE* Large zone emphasis, *HGZE* High grey-level zone emphasis

**Table 3:** Texture features that showed significantly different mean values comparing malignant *versus* benign solid lesions and corresponding area under the curve (AUC)

Feature	Malignant lesions (mean ± standard deviation)	Benign lesions (mean ± standard deviation)	*p* value	AUC (95% confidence interval)
Entropy	5.28 ± 0.38	5.67 ± 0.16	< 0.00001 for all	0.86 (0.82–0.89)
Skewness	0.74 ± 0.33	0.54 ± 0.41		0.66 (0.61–0.70)
Kurtosis	0.53 ± 0.67	0.31 ± 0.66		0.61 (0.56–0.65)
Contrast	24.45 ± 10.61	28.40 ± 13.66		0.58 (0.53–0.63)
GLN (GLCM)	96.33 ± 87.47	60.58 ± 61.73		0.67 (0.62–0.71)
RLN (GLCM)	2,218.97 ± 1,834.77	1,523.25 ± 1,681.38		0.66 (0.62–0.71)
HGRE (GLCM)	1,163.54 ± 20.49	1,171.63 ± 19.35		0.62 (0.57–0.67)
SRHGE (GLCM)	1,067.79 ± 32.58	1,080.79 ± 29.69		0.63 (0.58–0.67)
GLN (GLRLM)	96.43 ± 87.45	60.70 ± 61.72		0.67 (0.62–0.71)
RLN (GLRLM)	2,218.89 ± 1,828.26	1,525.31 ± 1,677.17		0.67 (0.62–0.71)
HGRE (GLRLM)	1,165.00 ± 20.59	1,173.06 ± 19.57		0.62 (0.57–0.67)
SRHGE (GLRLM)	1,069.20 ± 33.13	1,082.28 ± 29.75		0.62 (0.58–0.67)

*GLN* Grey-level non-uniformity, *GLCM* Grey-level co-occurrence matrix, *RLN* Run length non-uniformity, *HGRE* High grey-level run emphasis, *SRHGE* Short-run high grey-level emphasis, *GLN* Grey-level non-uniformity, *GLRLM* Grey-level run length matrix

### Machine learning

Correlation matrices for each sub-dataset (lesion *versus* tissue and benign *versus* malignant) with the FFS were displayed in Additional file 1: Figure S1A and S1B, respectively, showing significant co-correlation of several features among the higher-order features in A.

#### Sub-dataset (i): solid lesions versus normal tissue

**Fig. 4:**
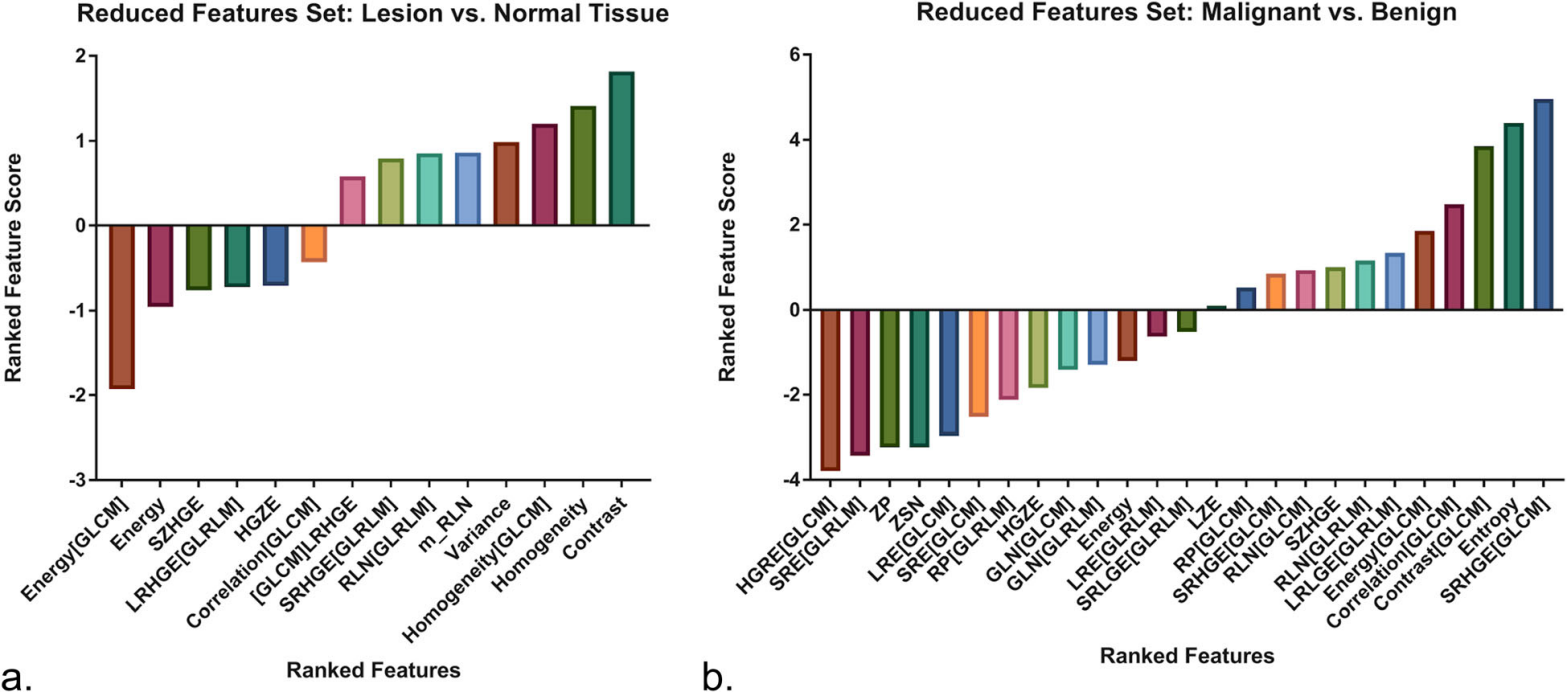
Ranked feature score in subset i (**a**) and in subset ii (**b**) (see text)

**Fig. 5:**
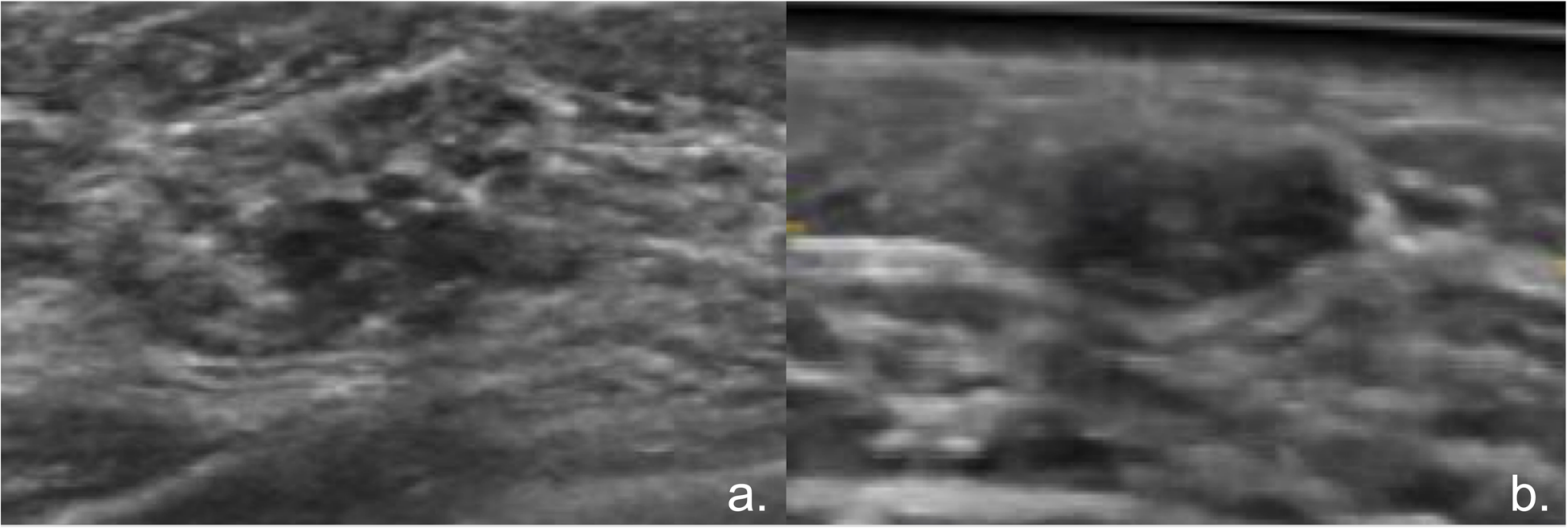
Lesions falsely classified as normal tissue using machine learning with the reduced feature set but correctly classified with the full feature set: invasive ductal carcinoma (maximal diameter 10 mm) in a 74-year-old patient (**a**) and fibroadenoma (maximal diameter 9 mm) in a 46-year-old patient (**b**). Lesion (**a**) was also falsely classified as benign in the comparison between malignant and benign lesions

The validation dataset included 105 images (54 images of lesions and 51 images of normal tissue). For the classification of lesions *versus* normal tissue, the optimal hyperparameters for the FFS accounted 1,000 and 0.001 for *C* and gamma, respectively (Additional file 1: Figure S2A). Classification accuracies of 92.8% on the training set and of 93.3 % on the validation set (Table 4) were reached, with 3.8% of all images in the validation partition being falsely classified as normal tissue and 2.9% as lesion instead of normal tissue (Table 5). ROC analyses revealed an AUC of 0.96 (95% CI 0.89–0.98) for the validation set (Fig. 2). After training and validating, the SVM classifier on the FFS, a recursive feature elimination with cross-validation, was performed determining 14 features (Fig. 4a) as optimal features, composing the RFS. For the RFS, a correlation matrix was generated and the optimal hyperparameters were determined as *C* = 1,000 and gamma = 0.00001 (Additional file 1: Figures S1C and S2B). Training and validation accuracies were 91.3% and 94.4%, respectively, with 1.9% of all images being falsely classified as lesions and 3.8% as normal tissue (Tables 4 and 5). The AUC for the RFS measured 0.98 (Fig. 2). For all showed texture feature-derived ROC curves (only features with AUC values equal or superior to 0.80) compared to the via ML-derived ROC curve, *p* values were < 0.05 (ranging from 0.003 to 0.02), indicating a significant difference between the areas. The two lesions incorrectly classified as normal tissue were one malignant and one benign (Fig. 5).

**Table 4:** Area under the curve (AUC), accuracy, sensitivity, and specificity achieved with the validation set in the classification of lesions *versus* normal tissue and malignant *versus* benign solid lesions using the full texture feature set and the reduced feature set

Sub-dataset	AUC (95% confidence interval)	Accuracy (%)	Sensitivity (%)	Specificity (%)
Lesions *versus* normal tissue				
Full feature set	0.96 (0.89–0.98)	93.3	92.6	94.1
Reduced feature set	0.98 (0.92–0.99)	94.4	96.3	92.1
Malignant *versus* benign lesions				
Full feature set	0.98 (0.81–0.99)	90.7	85.2	96.3
Reduced feature set	0.96 (0.90–0.99)	87.1	81.5	92.6

**Table 5:** Results in the validation set for the classification of lesions *versus* normal tissue and malignant *versus* benign solid lesions using the full texture feature and the reduced feature set

	Predicted		
Actual	Lesions *versus* normal tissue (*n* = 105)	Lesions (%)	Normal tissue (%)
	Lesions (*n* = 54)		
	Full feature set	50 (92.6)	4 (7.4)
	Reduced feature set	52 (96.3)	2 (3.7)
	Normal tissue (*n* = 51)		
	Full feature set	3 (5.9)	48 (94.1)
	Reduced feature set	4 (7.8)	47 (92.2)
	Malignant *versus* benign lesions (*n* = 54)	Malignant (%)	Benign (%)
	Malignant (tot = 27)		
	Full feature set	23 (85.2)	4 (14.8)
	Reduced feature set	22 (81.5)	5 (18.5)
	Benign (tot = 27)		
	Full feature set	1 (3.7)	26 (96.3)
	Reduced feature set	2 (7.4)	25 (92.6)

#### Sub-dataset (ii): malignant versus benign solid lesions

The validation dataset included 54 images (27 images of lesions and 27 images of normal tissue). For the classification of the malignant *versus* benign solid lesions, the optimal hyperparameters for the full feature set accounted 100 and 0.001 for *C* and gamma (Additional file 1: Figure S2C). The accuracy on the training set measured 89.0% and on the validation set 90.7% with 7.4% of all lesions being falsely classified as benign lesions and 1.9% falsely as malignant (Tables 4 and 5, Fig. 5). The AUC measured 0.98 (Fig. 3). After RFECV, a correlation matrix for the reduced feature set of 25 features (Fig. 4b) was generated applying the optimal hyperparameters of *C* = 1,000 and gamma = 0.001 (Additional file 1: Figures S1D and S2D). The classification accuracy for the RFS was 89.0% on the training and 87.1 % on the validation partition (Table 4). After feature reduction, the false-positive rate of malignant lesions being falsely classified as benign increased to 9.2 % and AUC decreased to 0.96 (Fig. 3). The ROC curve for entropy, derived from texture analysis, was significantly different (*p* = 0.003) from the via ML-derived ROC curve.

## Discussion

In the current study, we demonstrated that texture feature analysis of breast imaging findings in ABUS examinations might be used to differentiate malignant and benign solid lesions as well as normal tissue of the breast with high accuracy. We also showed that ML applied to texture data might be superior compared to the statistical analysis of single texture features.

Although the interrelation between the data derived from TA and potential underlying biological properties has not yet been resolved, a number of previous works have investigated the use of TA to quantify spatial heterogeneity of benign and malignant lesions in images acquired with different modalities [9–16]. A limited number of studies explored the use of TA or ML in ultrasound imaging for characterisation of breast lesions [28–30]. Indeed, the application of TA in conventional B-mode imaging is hindered by variations of scanning parameters that can determine unwanted variations in the assessment of texture features. Standardised acquisitions in ABUS can in part overcome these limitations.

In our study, a number of texture features exhibited significant differences when used to distinguish solid breast lesions from normal tissue as well as malignant from benign solid lesions with a relatively high AUC up to 0.86 in both cases. ML offers the possibility to train algorithms to recognise patterns of data derived from the analysis of multiple texture features instead of referring to a single feature. The use of a ML model based on the SVM algorithm with radial basis function determined an increase in the AUC to a maximum of 0.96 in the differentiation of lesions *versus* normal tissue as well as in the differentiation of malignant *versus* benign lesions with a maximal accuracy of 94.4% and 90.7%, respectively. The use of recursive feature selection in the test datasets for differentiation of lesions *versus* normal tissue resulted in an increase in the AUC to 0.98 whereas for malignant *versus* benign lesions, the AUC slightly decreased to 0.96. Moreover, application of the reduced feature sets resulted in nearly the same training accuracies for the training data and even a slightly higher accuracy of 94.4% for the test dataset differentiating lesions *versus* normal tissue. These excellent performances for the full as well as for the reduced feature sets and the associated low amount of overfitting emphasise the robustness and stability of the applied ML model. In many cases, overfitting occurs when the ML algorithm is trained in a too-large extent with details and noise negatively affecting the performance on real-world data. In order to minimise overfitting, the SVM on our study was trained via cross-validation, dividing the training data into subsets of equal size, which also provided advantages with respect to the limited number of data points. In addition, the robustness can be accounted, to some extent, that special interest was put into the acquisition of balanced datasets, and no oversampling techniques were applied to synthetically generate data [31].

Previous studies reported that the use of supplemental ABUS in breast cancer screening programmes causes an increase of the recall rate [6, 32]. Moreover, misinterpretation of lesions along with the presence of multiple distracting lesions are determining factors in the case of undiagnosed cancers at supplemental screening ultrasonography [33]. Although computer-aided-detection software for ABUS offers the potential to improve radiologists’ performances in detecting breast cancer, characterisation of the imaging findings remains a major issue [34, 35]. In a recent study, van Zelst et al. [35] showed that the AUCs between conventional ABUS reading and computer-aided-detection-based reading performed by eight radiologists with variable years of ABUS experience was not significantly different (0.82 and 0.83, respectively). The combined use of CAD software with algorithms, that enable TA combined to ML, might overcome the relative limitations of the two approaches (*i.e.,* the limited specificity of CAD and the necessity for aided-detection in TA combined to ML). Although the differentiation of breast lesions from normal breast tissue was quite straightforward in our cases, we decided to include also this evaluation considering the potential role of ML algorithms integrated in the software for ABUS image evaluation. A maximal accuracy of 94.4 was observed when comparing normal tissue *versus* breast lesions. More important, in our study, a very high specificity (maximal 96.3%) was achieved in the comparison of benign *versus* malignant lesions using ML.

Our study has some major limitations. First, the underpowered analysis due to the limited number of cases is included. Nevertheless, the purpose of our pilot study was to present a possible approach for the evaluation of breast imaging findings in ABUS and to enhance some differences when TA information alone or in conjunction with ML is used. A possibly prospective study including a higher number of cases is necessary to confirm our results. Second, the high number of evaluated images was derived from a relatively low number of different lesions that could have biased the results. Nevertheless, both malignant and benign solid lesions were collected from the general female population referred to our department for screening or follow-up examination of known lesions presumably forming a sufficient representative group of breast solid lesions. Third, we did not compare the performance of TA and ML with the performance of radiologists with different levels of experience, which was beyond the scope of this study. Also, although the inter-reader agreement for the assessment of the texture feature measurements was evaluated only in part of the lesions, we could demonstrate a high reproducibility of the measurements for all features.

In conclusion, our pilot study demonstrated that TA in combination with ML might represent a useful diagnostic tool in the evaluation of ABUS findings. Applying ML techniques to texture features might be superior compared to analysis of single texture features. A prospective study including a higher number of cases is necessary to confirm our results.

## Additional file

Additional file 1: **Table S1.** Inter-reader agreement for the different TA features was evaluated using the intraclass correlation coefficient (ICC). **Figure S1.** Correlation matrix generated from the full texture feature set for the sub-datasets lesions *versus* normal tissue (A) and malignant *versus* benign solid lesions (B) as well as from the corresponding reduced feature set (C) and (D). A significant co-correlation of several features is present in particular among the higher order features in A (*e.g.,* SRE[GLCM] and HGRE[GLCM]) as possible reflection of underlying common biological properties. **Figure S2.** Heatmaps depicting the optimal hyperparameters for the full feature (A, B) and the reduced feature training datasets (C, D). The hyperparameter tuning was implemented via nested grid search on the SVM classifier by specifying the parameter for gamma and (C) in a logarithmic scale from 0.00001 to 0.001 and 1 to 1000, respectively. (DOCX 4241 kb)

## Data Availability

The datasets used and/or analysed during the current study are available from the corresponding author on reasonable request.

## Abbreviations

ABUS: Automated breast ultrasound
AUC: Area under the curve
FFS: Full feature set
GLCM: Grey-level co-occurrence matrix
GLRLM: Grey-level run length matrix
GLSZM: Grey-level size zone matrix
HHUS: Hand-held ultrasound
ICC: Intraclass correlation coefficient
IQR: Interquartile range
ML: Machine learning
RFECV: Recursive feature elimination with cross-validation
RFS: Reduced feature set
ROC: Receiver operating characteristic
ROI: Region of interest
SVM: Support vector machine
TA: Texture analysis
