# Supplementary material for: Diagnostic performance of machine learning applied to texture analysis-derived features for breast lesion characterisation at automated breast ultrasound: a pilot study
Source: Eur Radiol Exp. 2019 Nov 1;3:44. doi: 10.1186/s41747-019-0121-6 (PMC6825080; doi:10.1186/s41747-019-0121-6)
Supplement: Supplementary file 1 — Table S1. Inter-reader agreement for the different TA features was evaluated using the intraclass correlation coefficient (ICC). Figure S1. Correlation matrix generated from the full texture feature set for the sub-datasets lesions versus normal tissue (A) and malignant versus benign solid lesions (B) as well as from the corresponding reduced feature set (C) and (D). A significant co-correlation of several features is present in particular among the higher order features in A (e.g., SRE[GLCM] and HGRE[GLCM]) as possible reflection of underlying common biological properties. Figure S2. Heatmaps depicting the optimal hyperparameters for the full feature (A, B) and the reduced feature training datasets (C, D). The hyperparameter tuning was implemented via nested grid search on the SVM classifier by specifying the parameter for gamma and (C) in a logarithmic scale from 0.00001 to 0.001 and 1 to 1000, respectively. (DOCX 4241 kb) [file 41747_2019_121_MOESM1_ESM.docx]

**Table S1.** Inter-reader agreement for the different TA features was evaluated using the intraclass correlation coefficient (ICC).

| Texture Features | ICC (95% confidence interval) | Texture Features | ICC (95% confidence interval) |
| --- | --- | --- | --- |
| Entropy | 0.90 (0.85-0.93) | SRE | 0.87 (0.54-0.94) |
| Variance | 0.79 (0.69-0.86) | LRE | 0.84 (0.51-0.93) |
| Skewness | 0.77 (0.56-0.87) | GLN | 0.88 (0.68-0.94) |
| Kurtosis | 0.82 (0.71-0.89) | RLN | 0.89 (0.75-0.95) |
| Contrast | 0.89 (0.76-0.94) | RP | 0.86 (0.53-0.94) |
| Correlation | 0.74 (0.60-0.83) | LGRE | 0.94 (0.91-0.96) |
| Energy | 0.94 (0.91-0.96) | HGRE | 0.67 (0.51-0.78) |
| Homogeneity | 0.88 (0.62-0.94) | m_SRLGE | 0.85 (0.78-0.90) |
| Contrast | 0.84 (0.72-0.91) | SRHGE | 0.74 (0.56-0.84) |
| Correlation | 0.86 (0.74-0.92) | LRLGE | 0.94 (0.91-0.96) |
| Energy | 0.84 (0.72-0.91) | LRHGE | 0.79 (0.53-0.89) |
| Homogeneity | 0.87 (0.58-0.94) | SZE | 0.89 (0.63-0.95) |
| SRE | 0.87 (0.56-0.94) | LZE | 0.80 (0.50-0.90) |
| LRE | 0.84 (0.52-0.93) | GLN | 0.67 (0.51-0.78) |
| GLN | 0.88 (0.68-0.94) | ZSN | 0.88 (0.63-0.95) |
| RLN | 0.89 (0.75-0.95) | ZP | 0.87 (0.55-0.94) |
| RP | 0.86 (0.54-0.94) | LGZE | 0.65 (0.48-0.77) |
| LGRE | 0.94 (0.90-0.96) | HGZE | 0.79 (0.66-0.86) |
| HGRE | 0.76 (0.61-0.85) | SZLGE | 0.82 (0.73-0.88) |
| SRLGE | 0.93 (0.89-0.96) | SHZGE | 0.78 (0.66-0.85) |
| SRHGE | 0.76 (0.61-0.85) | LZLGE | 0.70 (0.52-0.81) |
| LRLGE | 0.96 (0.94-0.97) | LZHGE | 0.69 (0.45-0.82) |
| LRHGE | 0.79 (0.52-0.89) | GLV | 0.75 (0.59-0.84) |
|  |  | ZSV | 0.73 (0.57-0.82) |

SRE: Short run emphasis; LRE: Long run emphasis; GLN: Gray-level non-uniformity; RLN: Run length non-uniformity; RP: Run percentage; LGRE: Low gray-level run emphasis; HGRE: High gray-level run emphasis; SRLGE: Short run low gray-level emphasis; SRHGE: Short run high gray-level emphasis; LRLGE: Long run low gray-level emphasis; LRHGE: Long run high gray-level emphasis; SRE: Short run emphasis; LRE: Long run emphasis; GLN: Gray-level non-uniformity; RLN: Run length non-uniformity; RP: Run percentage; LGRE: Low gray-level run emphasis; HGRE: High gray-level run emphasis; m_SRLGE: Short run low gray-level emphasis; SRHGE: Short run high gray-level emphasis; LRLGE: Log run low gray-level emphasis; LRHGE: Long run high gray-level emphasis; SZE: Small zone emphasis; LZE: Large zone emphasis; GLN: Gray-level non-uniformity; ZSN: Zone-size non-uniformity; ZP; Zone percentage; LGZE: Low gray-level zone emphasis; HGZE: High gray-level zone emphasis; SZLGE: Small zone low gray-level emphasis; SHGZE: small zone high gray-level emphasis; LZLGE: Large zone low gray level emphasis; LZHGE: Large zone high gray-level; GLV: Gray-level variance; ZSV: Zone size variance.


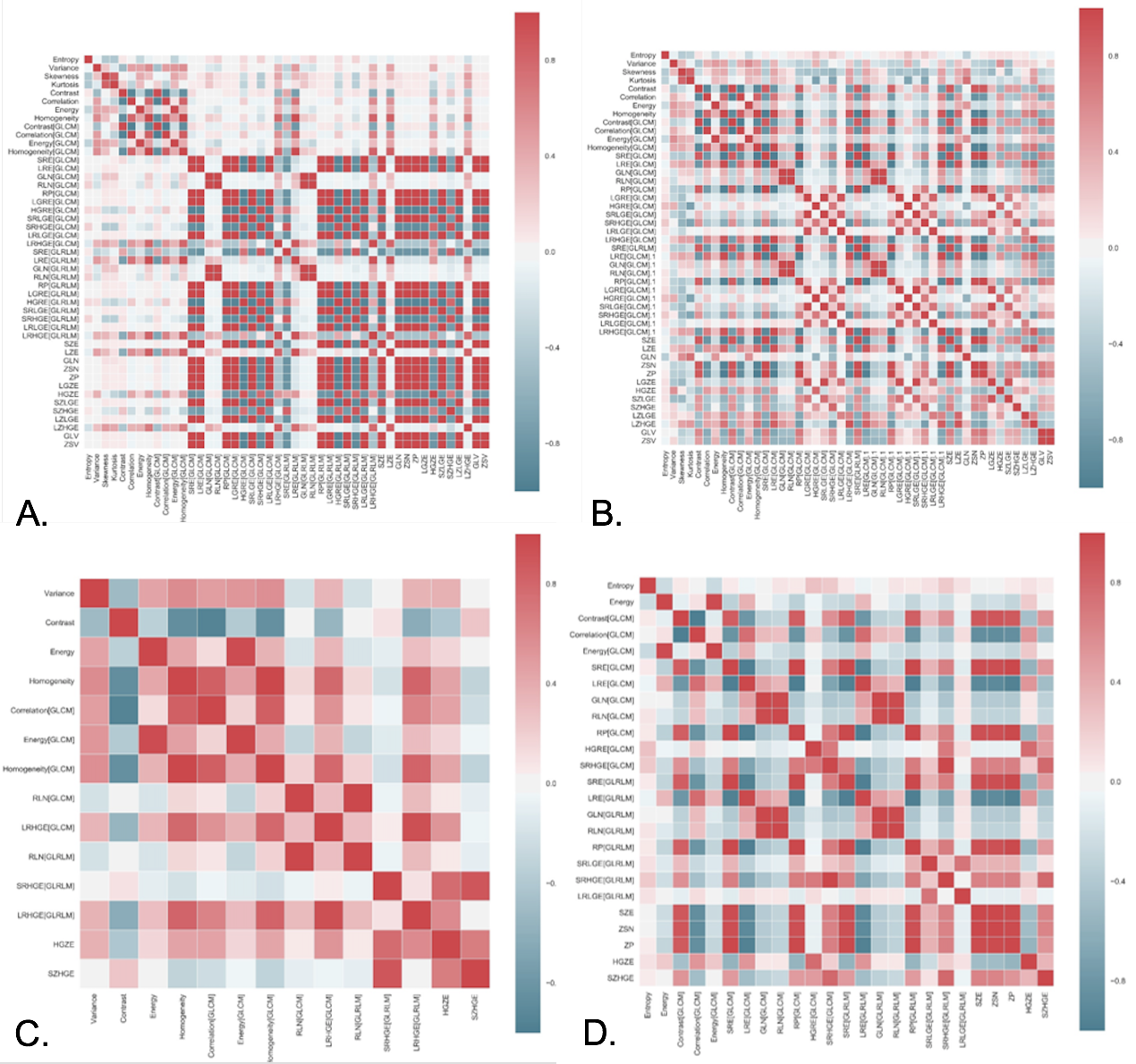


**Fig. S1** Correlation matrix generated from the full texture feature set for the sub-datasets lesions versus normal tissue (A) and malignant versus benign solid lesions (B) as well as from the corresponding reduced feature set (C) and (D). A significant co-correlation of several features is present in particular among the higher order features in A (e.g. SRE[GLCM] and HGRE[GLCM]) as possible reflection of underlying common biological properties.

**
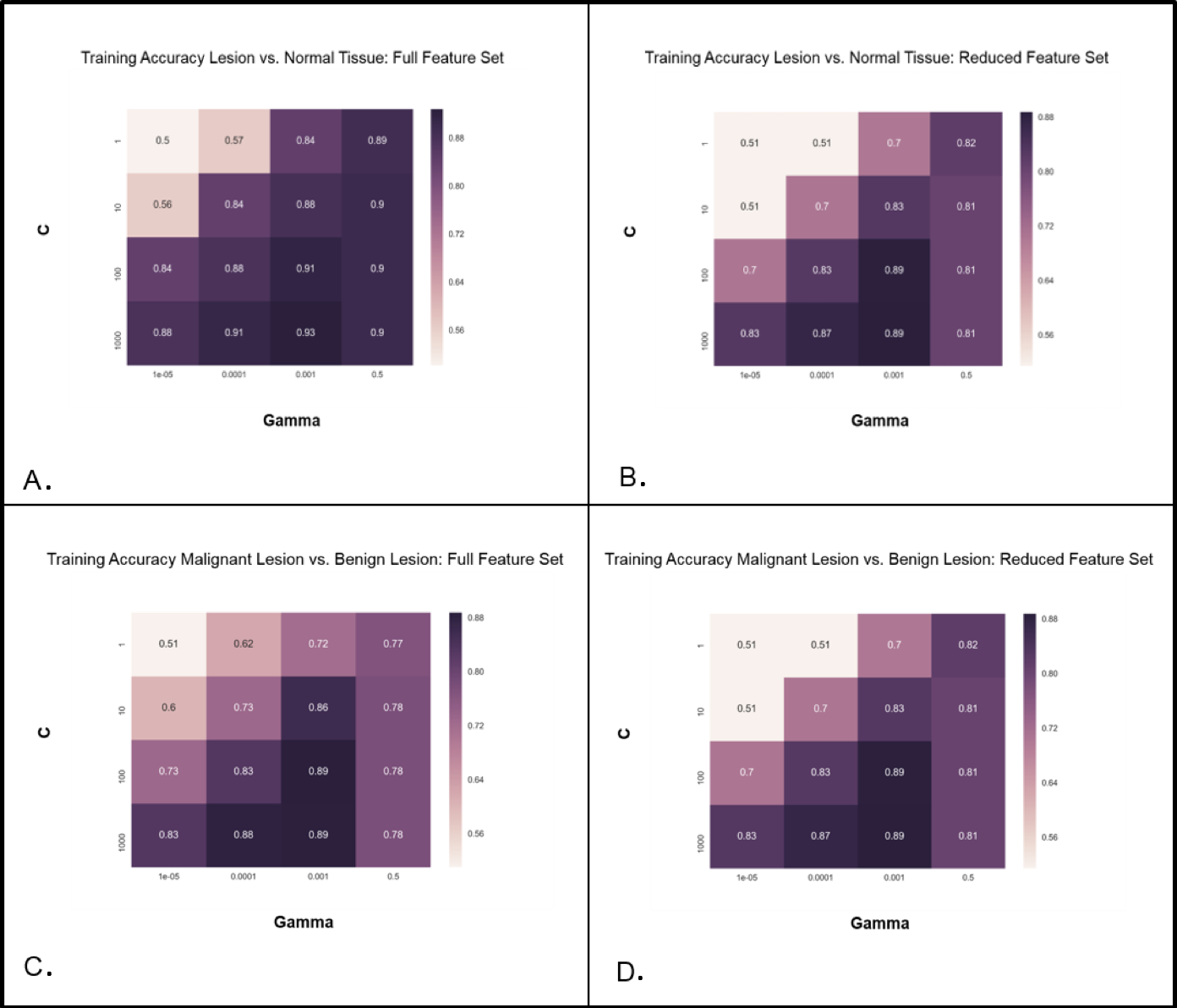
**

**Fig. S2** Heatmaps depicting the optimal hyperparameters for the full feature (A, B) and the reduced feature training datasets (C,D). The hyperparameter tuning was implemented via nested grid search on the SVM classifier by specifying the parameter for gamma and C in a logarithmic scale from 0.00001 to 0.001 and 1 to 1000, respectively.
